# Phenolic Composition and Antioxidant Activity of Plants Belonging to the *Cephalaria* (Caprifoliaceae) Genus

**DOI:** 10.3390/plants10050952

**Published:** 2021-05-11

**Authors:** Małgorzata Chrząszcz, Barbara Krzemińska, Rafał Celiński, Katarzyna Szewczyk

**Affiliations:** 1 Department of Pharmaceutical Botany, Medical University of Lublin, 1 Chodźki Str., 20-093 Lublin, Poland; malgorzata.chrzaszcz329@gmail.com (M.C.); barbara.krzem@gmail.com (B.K.); 2 Department of Cardiology, Independent Public Provincial Specialist Hospital in Chełm, 22-100 Chełm, Poland; rcelinski@op.pl

**Keywords:** *Cephalaria*, Caprifoliaceae, polyphenols, antioxidant activity

## Abstract

The genus *Cephalaria*, belonging to the Caprifoliaceae family, is a rich source of interesting secondary metabolites, including mainly saponins which display a variety of biological activities, such as immunomodulatory, antimicrobial and hemolytic effects. Besides these compounds, flavonoids and phenolic acids were identified in *Cephalaria* species. *Cephalaria* is employed in traditional medicine e.g., to cure cardiac and lung diseases, rheumatism, and regulate menstruation. In this review we focus on the phenolic compound composition and antioxidative activity of *Cephalaria* species. The antioxidant effect can be explained by flavonoids present in all parts of these plants. However, future efforts should concentrate more on in vitro and in vivo studies and also on clinical trials in order to confirm the possibility of using these plants as natural antioxidants for the pharmacology, food or cosmetic industries.

## 1. Introduction

Phenolic compounds are plentiful and ubiquitous secondary metabolites of plants [1] of great interest due to the fact that they are capable of preventing many diseases due to their antioxidant potential [2]. It is worth underlining that a significant enhancement of interest in the antioxidant properties of plants traditionally used in folk medicine has been observed [3], including rare or native wild species on which literature data was lacking [4]. The current focus is toward antioxidants of natural origin, therefore the number of publications on the subject of the favorable effects on health of plant polyphenols has boosted significantly [5]. Perron and Brumaghim have reported that several publications on radical scavenging activity by polyphenols has been released, representing more than 700 papers from 1995 till 2009 alone [6].

Many experiments carried out by various methods have shown that most of the antioxidant potential of plants result from the redox properties of their phenolic constituents [2,3]. Many mechanisms of action of antioxidants have been observed. Phenolic compounds may do the following: inhibit the formation of free radicals, enhance cellular antioxidant defense mechanisms, impair the action of pro-oxidative enzymes, neutralize pro-oxidant ions and boost other antioxidants’ action [7]. 

Bioactive phenolic compounds come from natural sources and simultaneously they are effective towards scavenging free radicals, which makes them very promising candidates for applications in health care, processed foods, the cosmetic industry and as auxiliary medicine remedies [3]. Thus, in recent years, the importance of the antioxidant activities of phenolic compounds and their potential usage in numerous kinds of industries as natural antioxidant compounds has reached a new level. Polyphenols are present in the human diet and are widely used for medical and cosmetic purposes [8]. The use of natural polyphenols in cosmetics is justified and worthwhile due to their capability to ameliorate cutaneous issues and applicability for antiaging purposes in cosmetics, as well as for nutraceutical applications. Natural polyphenols possess the potential to prevent premature ageing, decrease the occurrence of skin cancer, attenuate photoaging and protect skin against ultraviolet radiation [9]. Furthermore they have properties of depigmenting, impairing inflammation, healing wounds and mitigating skin irritation [7].

Phenolics have protective roles in many illnesses such as cancer, inflammation, cardiovascular and neurodegenerative diseases, which is recognized to be due to their potent antioxidant capacity. In addition to their antioxidant functions, polyphenols have many other biological activities, such as antihistamine, antiinflammatory, antiaging, antibacterial, antiviral, cardioprotective (increasing capillary resistance), hepatoprotective, anticancer (inducing apoptosis in cancer cells), antidiarrheal, neuroprotective properties (protecting neurological system), limiting weight gain, binding proteins such as caseins, inhibiting enzymes (telomerase α-amylase, pepsin, trypsin, and lipase), modulation of the immune system and promoting wound healing [1,6,10].

Taking the structure into consideration, the vast majority of polyphenols contain a tricyclic flavan ring system. Nevertheless, various structural differences are observed. Thus polyphenols encompass tremendous amounts of miscellaneous compounds, such as phenolic acids, flavonols, flavones, flavanols (catechins, epicatechins), flavanones, anthocyanidins, proanthocyanidins, isoflavones, flavanonols, stilbenes, coumarins, tannins, lignins, lignans, neolignans and antraquinones [6]. Flavonoids are the most plentiful, widely studied and also biologically active phytonutrients.

The qualitative and quantitative phenolic compound composition of plant extracts is determined by different factors, e.g., plant origin and habitat, plant development phase, seasonal variations and weather and climatic conditions. These secondary plant metabolites are produced in plants in response to various stresses, such as wounding, ultraviolet (UV) activity, infections, pollutants or ozone [7].

That having been said, this review was designed to comprehensively elucidate the relationship between the occurrence of phenolic compounds and antioxidant activity in *Cephalaria* Schrad. ex Roem. et Schult. species. The present study focuses on juxtaposing the species of this genus, in accordance with the obtained data.

The genus *Cephalaria* was previously placed in the Dipsacaceae Juss. family [11,12,13]. However, according to two latest versions of the system of the Angiosperm Phylogeny Group [14,15], the Dipsacaceae family belongs to the Caprifoliaceae Juss. s.l. According to new molecular phylogenetic and morphological research, *Cephalaria* is included in tribe Dipsaceae Rchb. of subfam. Dipsacoideae A. Eaton [13]. The genus encompasses approximately 95 species [16,17,18] that have been identified especially in the Mediterranean Basin and adjacent western Eurasia which are the major centers of biodiversity at a global level [19,20,21,22]. Several species occur also in Asia and eastern and southern Africa [13,23,24,25]. Plants belonging to the *Cephalaria* are annual, biennial or perennial herbs with glabrous or hairy stems and very variable leaves (most heterophyllous). Flowers are usually 4–partite, with a four angled, furrowed involucel, crowned with four angular hairy teeth, or with a membranous ± glabrous corona, entire, crenate or with four ± obtuse teeth [26].

Extracts from various species of *Cephalaria* have been used in traditional medicine for many years due to their antimicrobial, antifungal, cytotoxic, antioxidant, antidiabetic and hypothermic activities [27,28,29,30,31]. These plants are used in folk medicine to cure cardiac and lung diseases, rheumatism, and regulate menstruation [32,33]. Moreover, they are used in veterinary medicine and agriculture, as a wool dye and as an additive to bread [34,35]. Literature studies have shown that the *Cephalaria* species contain flavonoids [17,36,37], triterpenoid saponins [36,38,39,40], iridoids [36,37,40], alkaloids [37,41,42,43], lignans [37,44], fatty acids [45,46], that exhibit antioxidant, antimicrobial, cytotoxic, hemolytic, and immunomodulating activities [3,18,27,31,41,42].

## 2. Methodology of Evidence Aquisition

For a comprehensive literature overview, published phytochemical and antioxidant activity data were retrieved from the ISI^®^Web of Science, Scopus^®^, GoogleScholar^®^, SciFinder^®^, and Reaxys^®^ databases. Entries were considered until the end of March 2021. Exact spelling of scientific botanical names, including the abbreviations for botanical authors was brought in line with standard usage as recommended by “The International Plant Names Index” [47] and “The Plant List” [48]. Relevant original articles and books, with an unlimited time range and regardless of language were included in the review. Exclusion criteria were duplicate publications and non-relevant articles.

## 3. Phenolic Compounds in the *Cephalaria* Species

The investigations of *Cephalaria* species have led to the isolation and identification phenolic acids and different types of flavonoids, represented mostly by flavanone, flavonols, flavones, and anthocyanins. Table 1 summarizes such phenolic compounds (including the common/systematic name of constituent, species name and parts of the plant) mentioned in the surveyed literature.

### 3.1. Flavonoids

Flavonoids belong to a class of low-molecular-weight phenolics that are widely distributed in the plant kingdom. They have different subgroups, which include chalcones, flavones, flavonols, flavanones, flavan-3-ols, isoflavones and anthocyanidins [49].

In plants, flavonoids are responsible e.g., for the colour of flowers, the growth and development of seedlings. They also protect plants from various biotic and abiotic stresses and act as unique UV filters, allopathic compounds, and phytoalexins [49].

One of the first research on the occurrence of flavonoids in taxa of the *Cephalaria* genus dates from 1968, when Zemtsova and Bandyukova described the occurrence of quercetin 7-β-D-glucopyranoside (quercimeritrin) (**6**) in the aerial parts of *Cephalaria balkharica* E.A.Busch and in the flowers of. *C. gigantea* (Ledeb.) Bobrov. Moreover, luteolin 7-β-D-glucopyranoside (cynaroside) (**14**) was isolated from the aerial parts of *C. balkharica* and *C. gigantea* [50].

Nine years later, the same authors reported that flowers and leaves of *C. gigantea,* and *C. coriaceae* (Willd.) Roem. & Schult. ex Steud. contained genkwanin 6-C-β-D-glucopyranoside (swertisin) (**23**). In this study they also noted the presence of 7-O-methyluteolin 6-C-β-D-glucopyranoside (swertiajaponin, **15**) in the flowers and leaves of *C. uralensis* (Murray) Roem. & Schult. [51]. Furthermore, from the flowers of *C. kotschyi* Boiss. & Hohen. **6**, **14**, hyperoside (**9**) and kaempferol (**1**) were isolated [52].

From the 10% methanol extract from dried flowers of *C. pastricensis* Dörfl. & Hayek (from the Serbian-Bosnian border) **14** (15 mg) and luteolin 7-O-arabino(1 → 6)glucoside (**17**, 20 mg) were isolated [36]. 

Luteolin (**13**), quercetin (**5**), **6**, **14**, and a new flavonol bioside, namely gigantoside A (quercetin-7-O-[α-L-arabinopyranosyl(1→6)]-β-D-glucopyranoside) (**8**) were isolated from the flowers of *C. gigantea* (Republic of Azerbaijan) [53]. These authors found also that the flowers of *C. grossheimii* Bobrov (a synonym of *C. kotschyi*) contained **6**, **14**, apigenin (**12**), and hyperoside (**9**) [54]. Moreover, from the inflorescences of *C. procera* Fisch. et Avé-Lall. collected in Azerbaijan, Movsumov and co-authors extracted and identified compounds **5**, **6**, **8**, **12**, **13**, and **14** [55].

**Table 1:** The overview on the phenolic compounds identified in the *Cephalaria* genus.

Constituent Name	Species	Part of Plant	References
**1**. Kaempferol	*C. kotschyi*	aerial parts	Aliev and Movsumov, 1981 [52]
	*C. anatolica* *C. aristata* *C. aytachii* Göktürk & Sümbül *C. balansae* Raus *C. davisiana* Göktürk & Sümbül *C. elazigensis* var. *purpureaa* *C. elmaliensis* Hub.-Mor. & V.A.Matthews *C. isaurica* V.A.Matthews *C. lycica* V.A.Matthews *C. paphlagonica* Bobrov *C. procera* *C. scoparia* Contandr. & Quézel *C. speciosa* Boiss. & Kotschy *C. stellipilis* Boiss. *C. sumbuliana* Göktürk *C. taurica* Szabó *C. tuteliana* Kuș & Göktürk	aerial parts	Sarikahya et al., 2019 [56]
**2**. Astragalin	*C. anatolica* *C. aristata* *C. balansae* *C. davisiana* *C. elazigensis* var. *purpurea* *C. elmaliensis* *C. lycica* *C. paphlagonica* *C. procera* *C. speciosa* *C. stellipilis* *C. sumbuliana* *C. taurica*	aerial parts	Sarikahya et al., 2019 [56]
**3**. Nicotiflorin	*C. anatolica* *C. balansae* *C. paphlagonica* *C. speciosa* *C. stellipilis* *C. taurica*	aerial parts	Sarikahya et al., 2019 [56]
**4**. Tiliroside	*C. elmaliensis*	aerial parts	Sarıkahya and Kırmızıgül, 2012a [57]
**5**. Quercetin	*C. gigantea*	flowers	Movsumov et al., 2006 [53]
	*C. procera*	inflorescences	Movsumov et al., 2013 [55]
	*C. anatolica* *C. aristata* *C. balansae* *C. davisiana* *C. elazigensis* var. *purpurea* *C. isaurica* *C. lycica* *C. scoparia* *C. speciosa* *C. stellipilis* *C. taurica* *C. tchihatchewii* *C. tuteliana*	aerial parts	Sarikahya et al., 2019 [56]
**6**. Quercimeritrin	*C. balkharica*	aerial parts	Zemtsova and Bandyukova, 1968 [50]
	*C. kotschyi*	flowers	Aliev and Movsumov, 1981 [52]
	*C. gigantea*	flowers	Zemtsova and Bandyukova, 1968 [50] Movsumov et al., 2006 [53]
	*C. grossheimii*	flowers	Movsumov et al., 2009 [54]
	*C. procera*	inflorescences	Movsumov et al., 2013 [55]
**7**. Rutin	*C. gazipashensis*	aerial parts	Sarıkahya and Kırmızıgül, 2012 [31]
	*C. scoparia*	aerial parts	Sarikahya et al., 2015 [58]
	*C. anatolica* *C. aristata* *C. davisiana* *C. elmaliensis* *C. lycica*	aerial parts	Sarikahya et al., 2019 [56]
**8**. Gigantoside A	*C. gigantea*	flowers	Movsumov et al., 2006 [53]
	*C. procera*	inflorescences	Movsumov et al., 2013 [55]
**9**. Hyperoside	*C. kotschyi*	flowers	Aliev and Movsumov, 1981 [52]
	*C. grossheimii*	flowers	Movsumov et al., 2009 [54]
	*C. anatolica* *C. aristata* *C. aytachii* *C. balansae* *C. davisiana* *C. elazigensis* var. *purpurea* *C. elazigensis* var. *elazigensis* *C. elmaliensis* *C. isaurica* *C. lycica* *C. paphlagonica* *C. procera* *C. scoparia* *C. speciosa* *C. stellipilis* *C. sumbuliana* *C. taurica* *C. tchihatchewii* *C. tuteliana*	aerial parts	Sarikahya et al., 2019 [56]
	*C. uralensis*	aerial parts	Chrząszcz et al., 2020 [29]
**10**. Guiaverin	*C. lycica* *C. paphlagonica* *C. sumbuliana*	aerial parts	Sarikahya et al., 2019 [56]
**11**. Quercitrin	*C. gigantea*	roots	Tabatadze et al., 2017 [42] Tabatadze et al., 2020 [59]
**12**. Apigenin	*C. procera*	inflorescences	Movsumov et al., 2013 [55]
	*C. grossheimii*	flowers	Movsumov et al., 2009 [54]
	*C. aristata* *C. davisiana* *C. scoparia* *C. tchihatchewii*	aerial parts	Sarikahya et al., 2019 [56]
**13**. Luteolin	*C. gigantea*	flowers	Movsumov et al., 2006 [53]
	*C. procera*	inflorescences	Movsumov et al., 2013 [55]
	*C. anatolica* *C. aristata* *C. aytachii* *C. davisiana* *C. elazigensis* var. *elazigensis* *C. elmaliensis* *C. lycica* *C. scoparia* *C. sumbuliana* *C. taurica* *C. tchihatchewii* *C. tuteliana*	aerial parts	Sarikahya et al., 2019 [56]
**14**. Cynaroside	*C. balkharica*	aerial parts	Zemtsova and Bandyukova, 1968 [50]
	*C. gigantea*	aerial parts	Zemtsova and Bandyukova, 1968 [50]
	*C. kotschyi*	flowers	Aliev and Movsumov, 1981 [52]
	*C. pastricensis*	flowers	Godjevac et al., 2004 [36]
	*C. gigantea*	flowers	Movsumov et al., 2006 [53]
	*C. grossheimii*	flowers	Movsumov et al., 2009 [54]
	*C. procera*	inflorescences	Movsumov et al., 2013 [55]
	*C. elmaliensis*	aerial parts	Sarikahya et al., 2012a [57] Sarikahya et al., 2015 [58]
	*C. anatolica* *C. aristata* *C. aytachii* *C. balansae* *C. davisiana* *C. elazigensis* var. *purpurea* *C. elmaliensis* *C. isaurica* *C. lycica* *C. paphlagonica* *C. procera* *C. scoparia* *C. speciosa* *C. stellipilis* *C. sumbuliana* *C. taurica* *C. tchihatchewii* *C. tuteliana*	aerial parts	Sarikahya et al., 2019 [56]
**15**. Swertiajaponin	*C. uralensis*	flowers aerial parts	Chrząszcz et al., 2020 [27] Zemtsova and Bandyukova, 1977 [51]
	*C. isaurica*	aerial parts	Kayce and Kırmızıgül, 2010 [37]
	*C. elmaliensis*	aerial parts	Sarikahya et al., 2015 [58]
	*C. scoparia*	aerial parts	Sarikahya et al., 2015 [58]
	*C. gigantea*	aerial parts	Chrząszcz et al., 2020 [27]
**16**. Luteolin-7-*O*-rutinoside	*C. anatolica* *C. aristata* *C. elmaliensis* *C. isaurica* *C. lycica* *C. paphlagonica* *C. scoparia* *C. speciosa* *C. stellipilis* *C. sumbuliana* *C. taurica* *C. tchihatchewii* *C. tuteliana*	aerial parts	Sarikahya et al., 2019 [56]
**17**.Luteolin 7-*O*-arabino(1→6)glucoside	*C. pastricensis*	flowers	Godjevac et al., 2004 [36]
**18**. Diosmetin	*C. davisiana* *C. scoparia* *C. taurica* *C. tchihatchewii*	aerial parts	Sarikahya et al., 2019 [56]
**19**. Nepetin	*C. anatolica* *C. aristata* *C. aytachii* *C. balansae* *C. davisiana* *C. elazigensis* var. *purpurea* *C. elmaliensis* *C. isaurica* *C. lycica* *C. scoparia* *C. taurica* *C. tchihatchewii* *C. tuteliana*	aerial parts	Sarikahya et al., 2019 [56]
**20**. Isoorientin	*C. isaurica*	aerial parts	Kayce and Kırmızıgül, 2010 [37]
	*C. scoparia* *C. stellipilis*	aerial parts	Sarikahya et al., 2011 [43]
	*C. gigantea* *C. uralensis*	aerial parts aerial parts flowers	Chrząszcz et al., 2020 [27]
**21**. Isovitexin	*C. uralensis*	aerial parts flowers	Chrząszcz et al., 2020 [27]
**22**. Isovitexin *O*-hexoside	*C. gigantea*	aerial parts	Chrząszcz et al., 2020 [27]
**23**. Swertisin	*C. coriaceae*	flowers leaves	Zemtsova and Bandyukova, 1977 [51]
	*C. gigantea*	flowers leaves	Zemtsova and Bandyukova, 1977 [51]
**24**. Acacetin	*C. taurica*	aerial parts	Sarikahya et al., 2019 [56]
**25**. Hesperidin	*C. anatolica* *C. aristata* *C. aytachii* *C. davisiana* *C. lycica* *C. scoparia* *C. speciosa* *C. sumbuliana* *C. taurica* *C. tchihatchewii* *C. tuteliana*	aerial parts	Sarikahya et al., 2019 [56]
**26**. Genistein	*C. davisiana*	aerial parts	Sarikahya et al., 2019 [56]
**27**. Penduletin	*C. scoparia* *C. tuteliana*	aerial parts	Sarikahya et al., 2019 [56]
**28**. Cyanidin-3-*O*-glucoside	*C. anatolica* *C. aristata* *C. balansae* *C. davisiana* *C. elazigensis* var. *purpurea* *C. elazigensis* var. *elazigensis* *C. elmaliensis* *C. lycica* *C. paphlagonica* *C. procera* *C. scoparia* *C. speciosa* *C. stellipilis* *C. sumbuliana* *C. tchihatchewii*	aerial parts	Sarikahya et al., 2019 [56]
**29**. Pelargonidin chloride	*C. aristata* *C. davisiana* *C. speciosa*	aerial parts	Sarikahya et al., 2019 [56]
**30**. Chlorogenic acid	*C. syriaca*	shoot	Ali et al., 2012 [17]
	*C. ambrosioides*	roots	Pasi et al., 2002 [60]
	*C. gigantea*	roots	Tabatadze et al., 2017 [42] Tabatadze et al., 2020 [59]
	*C. uralensis*	aerial parts flowers	Chrząszcz et al., 2020 [27]
**31**. Cryptochlorogenic acid	*C. gigantea*	aerial parts	Chrząszcz et al., 2020 [27]
	*C. uralensis*	aerial parts flowers	Chrząszcz et al., 2020 [27]
**32**. Neochlorogenic acid	*C. gigantea*	aerial parts	Chrząszcz et al., 2020 [27]
	*C. uralensis*	aerial parts flowers	Chrząszcz et al., 2020 [27]
**33**. 3,5-*O*-dicaffeoylquinic acid	*C. ambrosioides*	roots	Pasi et al., 2002 [60]
	*C. gigantea*	aerial parts	Chrząszcz et al., 2020 [27]
	*C. uralensis*	aerial parts flowers	Chrząszcz et al., 2020 [27]
**34**. 4,5-*O*-dicaffeoylquinic acid	*C. gigantea*	aerial parts	Chrząszcz et al., 2020 [27]
	*C. uralensis*	aerial parts flowers	Chrząszcz et al., 2020 [27]
**35**. 3,4-di-*O*-caffeoylquinic acid	*C. ambrosioides*	roots	Pasi et al., 2002 [60]
**36**. Caffeic acid	*C. gigantea*	roots	Tabatadze et al., 2017 [42] Tabatadze et al., 2020 [59]
	*C. gigantea*	aerial parts	Chrząszcz et al., 2020 [27]
	*C. uralensis*	aerial parts flowers	Chrząszcz et al., 2020 [27]
	*C. anatolica* *C. aristata* *C. aytachii* *C. balansae* *C. davisiana* *C. elazigensis* var. *purpurea* *C. elazigensis* var. *elazigensis* *C. elmaliensis* *C. isaurica* *C. lycica* *C. paphlagonica* *C. scoparia* *C. speciosa* *C. stellipilis* *C. sumbuliana* *C. taurica* *C. tchihatchewii* *C. tuteliana*	aerial parts	Sarikahya et al., 2019 [56]
**37**. Ferulic acid	*C. uralensis*	aerial parts	Chrząszcz et al., 2020 [27]
**38**. Gallic acid	*C. syriaca*	seeds	Ali et al., 2012 [17]
**39**. *p*-Hydroxybenzoic acid	*C. syriaca*	roots seeds	Ali et al., 2012 [17]
**40**. *trans*-4-OH-Cinnamic acid	*C. aristata* *C. davisiana*	aerial parts	Sarikahya et al., 2019 [56]
**41**. Sinapic acid	*C. syriaca*	seeds	Ali et al., 2012 [17]
**42**. Syringic acid	*C. syriaca*	seeds shoots	Ali et al., 2012 [17]
**43**. Vanillic acid	*C. syriaca*	seeds shoots	Ali et al., 2012 [17]

Kayce and Kırmızıgül [37] isolated two flavone *C*-glycosides, swertiajaponin (**15**, 194.0 mg) from *C. isaurica* V.A. Matthews and isoorietin (**20**, 23.7 mg) from the butanol extract of the aerial parts of *C. stellipilis*. Isoorientin (**20**) was also isolated from the aerial parts of *C. scoparia* (57.4 mg obtained from 36.0 g of *n*-BuOH extract) and *C. stellipilis* (23.7 mg obtained from 34.3 g of *n*-BuOH extract) [43].

A flavone glycoside, rutin (**7**), was identified in the aerial parts of *C. gazipashensis* Sümbül collected from Antalya Province (Turkey) [31] and in the aerial parts of *C. anatolica* Shkhiyan, *C. aristata* K. Koch, *C. davisiana*, *C. elmaliensis* Hub.-Mor. & V.A. Matthews, and *C. lycica* [56]. This compound was also isolated from the *C. scoparia* acetone extract (3.1 mg) [58]. From the aerial parts of *C. elmaliensis* cynaroside (**14**, 2.4 mg) [58] and tiliroside (kaempferol 3-*O*-β-D-(6″-*O*-(*E*)-*p*-coumaroyl)glucopyranoside, **4**) were isolated [57]. Quercitrin was identified in the ethyl acetate and aqueous fractions from the roots of *C. gigantea* [42,59].

Twenty five flavonoids were measured simultaneously in the aerial parts of nineteen *Cephalaria* species [56]. The authors found that the main flavonoids in the studied plants were **5** (0.05–5.47 mg/g), **9** (0.01–7.65 mg/g), **13** (0.01–4.45 mg/g), **14** (0.02–4.91 mg/g), hesperidin (**25**, 0.11–29.79 mg/g), cyanidin-3-*O*-glucoside (**28**, 0.07–20.59 mg/g), and astragalin (kaempferol-3-*O*-glucoside, **2**, 0.16–9.27 mg/g). The other flavonoids identified in this study were **1** (0.01–0.44 mg/g), **7** (0.13–0.60 mg/g), **12** (0.01–1.33 mg/g), nicotiflorin (kaempferol-3-*O*-rutinoside, **3**, 0.19–1.06 mg/g), guiaverin (quercetin-3-*O*-arabinoside, **10**, 0.11–0.72 mg/g), luteolin-7-*O*-rutinoside (**16**, 0.03–0.61 mg/g), diosmetin (luteolin 4′-methyl ether, **18**, 0.01–0.28 mg/g), nepetin (6-methoxyluteolin, **19**, 0.01–1.26 mg/g), acacetin (5,7-dihydroxy-4′-methoxyflavone, **24**, 0.02 mg/g), genistein (4′,5,7-trihydroxyisoflavone, **26**, 0.18 mg/g), penduletin (4′,5-dihydroxy 3,6,7-trimethoxyflavone, **27**, 0.01 mg/g), pelargonidin chloride (**29**, 0.06–0.65 mg/g). Moreover, the highest flavonoid content was found in the aerial parts of *C. tchihatchewii* Boiss. (from 0.08 to 29.79 mg/g). The extract of *C. davisiana* contained secondly high level of flavonoids (from 0.02 to 14.78 mg/g). Besides that, the most abundant flavonoids—**25** (29.79 mg/g) and **28** (20.59 mg/g) were detected in *C. tchihatchewii* among all studied species.

In the latest study from 2020, Chrząszcz et al., reported that the aerial parts of *C. uralensis* contained **9** (0.86 μg/g of dry extract)**, 20** (41.71–65.18 μg/g of dry extract) and **21** (1.87–4.67 μg/g of dry extract), and the flowers—**15** (7.91–40.19 μg/g of dry extract), **20** (48.50–51.72 μg/g of dry extract) and **21** (4.20 μg/g of dry extract). Moreover, in the aerial parts of *C. gigantea* **15** (80.45–115.10 μg/g of dry extract), **20** (79.15–108.42 μg/g of dry extract) and **22** (2.15–2.98 μg/g of dry extract) were identified using LC-DAD-MS/MS method [27].

### 3.2. Phenolic Acids

Phenolic acids are a large group of phenolic compounds in plants, that include two groups—hydroxybenzoic (C_6_-C_1_ structures; e.g., gallic, *p*-hydroxybenzoic, protocatechuic, syringic) and hydroxycinnamic (C_6_-C_3_ structures; e.g., caffeic, ferulic, synapic) acid derivatives with various number and position of methoxylation and hydroxylation in aromatic ring. In plants, these compounds exist in their free and bound forms, and more often bound forms occur as their glycosides and esters [59]. Phenolic acids have a crucial for plants growth and reproduction, and they are produced as a response to environmental factors (e.g., light) and to defend injured plants [61]. What is more, they are reported to have a wide spectrum of pharmacological activities including antioxidant [62], antibacterial [63], anti-inflammatory [64], and anticarcinogenic [59] activities.

To date, there are only a few reports regarding the occurrence of phenolic acids of the *Cephalaria* genus. The most frequently identified phenolic acid is caffeic acid (**36**), which was found in the roots of *C. gigantea* [42,59], aerial parts (0.84–1.27 μg/g of dry extract) and flowers (0.79–0.91 μg/g of dry extract) of *C. uralensis* [18] and in the aerial parts of eighteen species (0.01–4.27 mg/g) collected in the Anatolia area (Turkey) [46].

In the aerial parts of *C. gigantea* chlorogenic acid (**30**, 101.79–135.83 μg/g of dry extract), cryptochlorogenic acid (**31**, 16.02–20.80 μg/g of dry extract), neochlorogenic acid (**32**, 5.13–9.35 μg/g of dry extract), 3,5-*O*-dicaffeoylquinic acid (**33**, 73.53–118.90 μg/g of dry extract) and 4,5-*O*-dicaffeoylquinic acid (**34**, 11.57–13.43 μg/g of dry extract) were detected [27], and in the roots of this species **30** and **36** was identified [42,59]. Moreover, the authors found that higher concentration of phenolics (phenolic acids and flavonoids) was contained in the ethyl acetate fraction from the roots of *C. gigantea* than in the aqueous fraction [42,59].

Chrząszcz et al. [27] identified in the aerial parts and flowers of *C. uralensis* **30** (114.90–132.18 and 94.90–98.75 μg/g of dry extract, respectively), **31** (1.68–4.01 and 7.45 μg/g of dry extract, respectively), **32** (3.87–8.75 and 3.42–8.54 μg/g of dry extract, respectively), **33** (58.35–70.26 and 41.29–48.30 μg/g of dry extract, respectively), and **34** (8.07–17.81 and 7.18–7.65 μg/g of dry extract, respectively).

Ali and co-authors [17] investigated different *C. syriaca* parts and they described the occurrence of **30**, syringic acid (**42**) and vanillic acid (**43**) in the shoots, gallic acid (**38**), *p*-hydroxybenzoic acid (**39**), sinapic acid (**41**), **42** and **43** in the seed, and **39** in the roots.

Three hydroxycinnamic esters—**30**, 3,4-di-*O*-caffeoylquinic acid (**35**) and **33—**were isolated from the roots of *C. ambrosioides* collected in Athens (Greece). All these compounds were identified using spectral data [60].

### 3.3. Antioxidant Activity

Most of the antioxidant potential in plants is caused by the redox properties of phenolic compounds that make it possible for them to act as hydrogen donors, reducing agents, and singlet oxygen quenchers. Their antioxidant activity is a result of different mechanisms such as free radicals scavenging, metal ion chelation, reduction, oxidase inhibition, as cofactors of enzymes catalyzing oxidative reactions, free radical stabilization and radical chain reaction termination [62,65,66].

The antiradical activity of the flavonoids isolated from the flowers of *C. pastricensis* was evaluated using the 1,1-diphenyl-2-picrylhydrazyl (DPPH) radical scavenging assay. It was found that cynaroside (**14**) and luteolin 7-*O*-arabino(1 → 6)glucoside (**17**) in different concentrations (5–80 μM) possess significant antiradical activity with EC_50_ = 41.3 μM and 41.4 μM, respectively [36].

The antioxidant activity of compounds isolated from the aerial parts of *C. isaurica*, *C. paphlagonica*, *C. scoparia*, and *C. stellipilis* was evaluated using the DPPH radical scavenging and CUPric Ion Reducing Antioxidant Capacity (CUPRAC) methods. The authors found that isoorientin was the most effective antioxidant compound in both the DPPH (IC_50_ = 0.119 ± 0.0004 mg/mL, while for ascorbic acid was 0.01 ± 0.002 mg/mL) and CUPRAC (6.683 ± 0.636 mmol TRg^−1^) assyas, with a value comparable to Trolox and ascorbic acid used as the positive controls [43]. Isoorientin is well known antioxidant and its structure-activity relationship is well documented [43,67].

Kirmizigül et al., evaluated *n*-hexane extracts of *C. davisiana*, *C. elazigensis*, *C. paphlagonica* and *C. stellipilis* from different regions of Turkey, using the CUPRAC assay, for the cupric (II) reducing antioxidant capacity, were 0.334, 0.252, 0.136 and 0.120 mmol TR/g dry extract, respectively. It seems that antioxidant activity of these species resulted from synergistic effect of ALA and phytol. The extracts exhibited a high antioxidative activity of 0.334–0.120 mmol TR/g dry extract. *C. davisiana* was the most effective cupric (II) reducer [68].

Sarıkahya and co-authors tested also the hexane extracts of ten *Cephalaria* species (*C. anatolica*, *C. aristata*, *C. aytachii*, *C. elazigensis* var. *elazigensis*, *C. hirsuta Stapf*, *C. taurica*, *C. tuteliana*, *C. procera*, *C. speciosa*, *C. tchihatchewii*) for their antioxidant capacity using the DPPH radical scavenging and CUPRAC methods. The DPPH tests revealed that hexane extracts of *C. tchihatchewii*, *C. hirsuta*, *C. anatolica*, *C. elazigensis* var. *elazigensis* and *C. speciosa* have significant radical scavenging activity, with the IC_50_ values of 3.77 ± 0.67, 5.13 ± 1.04, 5.20 ± 0.92, 5.28 ± 0.46 and 6.17 ± 3.13 mg/mL, respectively. The highest TEAC value (1.005 mmol ± 0.13 TE/g extract) they found for *C. aristate* and its reducing power was related to phenolic content (2.91 ± 0.15 mg GAE/g extract). The authors concluded that DPPH scavenging potential of *Cephalaria* extracts may be attributed to their phenolic compounds, that could donate electrons to DPPH. Because in the CUPRAC method, the reactive -OH groups of phenolic antioxidants are oxidized to the corresponding quinones and Cu(II)-bis(neocuproine) is reduced to the chelate, Cu(I)-bis(neocuproine), the correlation between CUPRAC values and phenolic contents of *C. tchihatchewii*, *C. aristata* and *C. speciosa* in this study is consistent with the above phenomenon [45]. 

Mbhele et al., evaluated various extracts (acetone, ethanol, methanol, hydroethanol and water) of the leaves and roots of *C. gigantea* by means of three different assays, including the DPPH radical test, 2,2′-azinobis[3-ethylbenzthiazoline]-6-sulfonic acid (ABTS^•+^) decolorization test, and the ability to reduce FeCl_3_ solution. Water extract from the leaves and roots possessed the lowest IC_50_ (0.6 and 2.8 μg/mL, respectively) in the DPPH assay. Hydroethanolic extract from the leaves had the lowest IC_50_ for both ABTS radical scavenging (1.0 μg/mL) and reducing activity (1.7 μg/mL). The water and hydroethanolic extracts of both leaves and roots of *C. gigantea* contained the highest amounts of phenolics and flavonoids and this suggest that these compounds could be responsible for their strong antioxidant activity [28].

The antioxidant activity of a *C. jopponsis* aqueous, ethanolic and ethyl acetate extracts were evaluated in vitro (phosphomolybdenum method) [38]. The studied extracts showed antioxidant activity ranging from 20.7 to 41.1 mg of ascorbic acid/g dry extract. Furthermore, Rahimi and co-authors [69] studied the effect of various fertilizers on the antioxidant activity of *C. syriaca* and they concluded that the antioxidant capacity (DPPH assay) of the studied samples was ranging from 47.10–60.16%.

Kavak and Baştürk [34] analyzed the antioxidant activity of the seeds of *C. syriaca* collected from different areas in Turkey. They found that studied extracts possessed DPPH inhibition activity ranging from 18.8 to 67.3%. Moreover, the ABTS results (TEAC values) were demonstrated values from 9.8 to 41.8 mmol Trolox eq/g DW.

The antioxidant activity of the oil extracted from the seeds of *C. syriaca* was evaluated by Atalan et al. [70]. The authors found that in the DPPH^•^ test, plant extracts did not have a high activity. The highest value was observed at 70 μL/mL concentration and it was 9.27 μL/mL while the percent of DPPH inhibition by ascorbic acid (used as a standard substance) was 83.75 μL/mL.

The antioxidant effect of *C. gigantea* and *C. uralensis* extracts were evaluated in vitro using DPPH^•^, ABTS^•+^ and metal chelating assays. The higher DPPH^•^ scavenging activity was found for the aerial parts of *C. uralensis* (IC_50_ = 2.86 ± 0.12 mg/mL). The extract from the flowers of *C. uralensis* demonstrated the highest scavenging free radical effect in the ABTS^•+^ (IC_50_ = 0.45 ± 0.21 mg/mL). The extracts from the aerial parts of *C. uralensis* were also the most active ones interfering with the formation of iron and ferrozine complexes, that suggest their high chelating capacity [27]. The main compounds identified in these extracts were chlorogenic acid (**30**), isoorientin (**20**) and swertiajaponin (**15**), the compounds which are well-known natural antioxidants showing strong effects in different tests [71].

### 3.4. Conclusions and Research Gaps/Future Investigations

This review summarizes the phenolics contain and antioxidant activity of species of the *Cephalaria* genus. According to literature information, only 29 species of the genus have been studied so far, and the available data are still fragmentary and insufficient. Moreover, the state of knowledge of *Cephalaria* species contains some gaps, which require more investigation.

So far, in the *Cephalaria* species, only 43 compounds belonging to the phenolic acids and flavonoids classes have been identified. Kaempferol, luteolin and quercetin and its derivatives have been the major constituents found in the investigated species. What is more, most of phenolic compounds they were detected using old, not very precise methods. Thus, it would be advisable to reexamine *Cephalaria* species for the presence of these compounds using modern analytical methods.

It seems to be interesting to combine these results with those of a chemotaxonomic study to see if there is any correlation between chemical profile and molecular and/or morphological features.

All the abovementioned findings suggest that an obvious gap in our knowledge about the *Cephalaria* genus also concerns their antioxidant activity. The research carried out so far has shown that these plants have a strong antioxidant potential. Thus, a focused investigation of the other species, and compounds isolated might be helpful to identify possible uses of these plants in the pharmacology, food or cosmetic industries.

## Data Availability

MDPI Research Data Policies.
